# Asymmetric properties of rod cGMP Phosphodiesterase 6 (PDE6): structural and functional analysis

**DOI:** 10.1186/2050-6511-16-S1-A76

**Published:** 2015-09-02

**Authors:** Bilal M Qureshi, Elmar Behrmann, Johannes Schöneberg, Justus Loerke, Jörg Bürger, Thorsten Mielke, Jan Giesebrecht, Frank Noé, Klaus Peter Hofmann, Christian M T Spahn, Martin Heck

**Affiliations:** 1 Biophysics and Medical Physics, Charité – Universitätsmedizin Berlin, 10117 Berlin, Germany; 2 Department of Mathematics, Computer Science and Bioinformatics, Freie Universität Berlin, 14195 Berlin, Germany; 3 Microscopy & Cryo Electron Microscopy Group, Max-Planck Institut für Molekulare Genetik, 14195 Berlin, Germany; 4 Zentrum für Biophysik und Bioinformatik, Humboldt-Universität zu Berlin, 10115 Berlin, Germany; 5 Present address: Research Group Structural Dynamics of Proteins, Center of Advanced European Studies and Research (caesar), 53175 Bonn, Germany; 6 Present address: Department of Molecular & Cell Biology, University of California at Berkeley, Berkeley, CA 94720, USA; 7 Present address: FEI VSG (Visualization Science Group), Zuse Institut Berlin, 14195 Berlin, Germany

## 

Photoreceptor cGMP Phsophodiesterase 6 (PDE6) is the effector molecule of visual signal transduction and mediates fast response of light signals. The rod holo-PDE6 comprises catalytic (α, β; each ~ 90 kDa) and two identical inhibitiory (γ; ~ 10 kDa) subunits. The catalytic subunits comprise N-terminal tandem GAF domains followed by C-terminal catalytic domains and isoprenylations for membrane-association. Contrary to activation of other tandem GAF comprising PDEs, PDE6 activation does not occur via cGMP-induced concerted conformational changes. Rather two copies of the α-subunit of retinal G-Protein (Gα*), transducin, activate PDE6 by partially displacing the inhibitory subunits. The activation of PDE6 has therefore been described as a “de-inhibition”. The affinity of Gα* to PDE6 and the enzymatic activity of the intermediary 1:1 complex is highly disputed, therefore a conclusive activation model is lacking so far.

Our combined structural, enzymatic and computational investigations deal with the activation-mechanism of PDE6. Our cryo electron microscopy (EM) structure of PDEαβ catalytic core shows an elongated bell-shaped structure with symmetric side-by-side arrangement of the two subunits with flexible membrane-binding domains. A comparison with nearly full-length inactive PDE2A structure [1] suggests that less compaction of both subunits and higher degree of conformational freedom of the catalytic domains result in constitutive activation of PDE6αβ, which is kept inactive by the inhibitory γ subunits. Furthermore, the structure of PDE6 suggests Gα* binding-sites pointing to opposing faces. The enzymatic characterization using Gα* titration of the PDE6 however reveal striking asymmetry of the two catalytic subunits with a high and a low affinity binding site for Gα*. Occupancy of the PDE6 with one Gα* induces negligible activity, whereas occupancy with two copies of Gα* leads to full enzyme activity. Such an activation mechanism constitutes a “coincidence switch” that allows noise filtering (i.e., spontaneously produced Gα* do not activate PDE6). Our spatiotemporal simulation work indeed confirms that spontaneously generated Gα* lead to the formation of singly occupied PDE6 and only a high local concentration of Gα*, as produced by an active receptor (rhodopsin), leads to doubly Gα* occupied effector complex. Therefore the localized large concentration of Gα* combined with the asymmetric properties of PDE6 constitutes a “density switch” that allows suppression effector level noise and reliable reporting of single quantum events in rod photoreceptor cells.

## References

[B1] Pandit J Forman MD Fennell KF Dillman KS Menniti FS Mechanism for the allosteric regulation of phosphodiesterase 2A deduced from the X-ray structure of a near full-length construct PNAS 2009 106 18225 18230 10.1073/pnas.0907635106 19828435 PMC2775329

